# Evaluation of cytotoxicity of different root canal sealers and their effect on cytokine production

**Published:** 2009-01-07

**Authors:** Ali Kangarloo, Mandana Sattari, Faranak Rabiee, Seyed Omid Dianat

**Affiliations:** 1 *Department of Endodontics, Iranian Center for Endodontic Research, Dental Research Center, Dental School, Shahid Beheshti University of Medical Sciences, Tehran, Iran*; 2 *Department of Immunology, Shahid Beheshti University of Medical Sciences, Tehran, Iran*; 3 *Endodontist, Tehran, Iran*; 4 *Department of Endodontics, Iranian Center for Endodontic Research, Dental School, Shahid Beheshti University of Medical Sciences, Tehran, Iran*

**Keywords:** Cytotoxicity, Endodontics, Fibroblast, Interleukin-6, L929, Sealers

## Abstract

**INTRODUCTION:** Endodontic sealers are in direct contact with periradicular tissues and play a critical role in regeneration and pathogenesis of periradicular diseases. The aim of this study was to compare cytotoxicity of four different types of sealers including AH plus, Sankin, Tubliseal EWT and Apexit as well as their effect on cytokine release of L929 fibroblasts.

**MATERIALS AND METHODS:** In this experimental study, cells were cultured in Complete Medium Culture (CMC) and then divided into two test groups. In group 1, sealers were added to cell culture wells immediately after mixing. In group 2, sealers were added to cell cultures 3 hours after mixing. Cell viability was evaluated by MTT assay after 4, 24 and 168 hours. The amount of Interleukin-6 (IL-6) released in response to the sealers was also evaluated by ELISA technique on fibroblasts after 24 hour period. Data were analyzed using Kruskal-Wallis and Mann Whitey-U tests.

**RESULTS:** Significant differences were seen in cytotoxicity in both groups (P<0.001). The least cytotoxic sealers were AH Plus and Sankin respectively, whereas Tubliseal EWT showed the greatest cytotoxicity. Production of IL-6 was significantly different among studied groups (P<0.001). The highest IL-6 level was observed in Tubliseal EWT and Sankin groups; which was statistically significant (P<0.001).

**CONCLUSION:** AH plus has less cytotoxicity and induces less IL-6 release. Tubliseal EWT has greater cytotoxicity and induces more IL-6 release than other sealers. This should be considered during their routine use in root canal treatments.

## INTRODUCTION

When considering the effect of sealers on regeneration of adjacent periradicular tissues, their importance in root canal obturation is undeniable. Studies have shown abundant toxic and inflammatory effects for sealers. Manufactures have claimed that the improved properties of new sealers have resulted in lower cytotoxicity, that is toxic agents have been removed, however controversies still exist with current research (1).

According to Kouluouzidou *et al.*, AH26 had higher cytotoxicity than AH Plus and Topseal (2). Leyhausen *et al.* evaluated the cytotoxic and genotoxic effects of AH Plus sealer and showed that AH Plus had very low cytotoxicity (3). Gheshlaghi *et al.* studied cytotoxic effects of AH Plus, AH26 and ZOE sealers on human gingival fibroblasts and demonstrated that ZOE had a high cytotoxicity which remain constant even after 5 weeks, whereas AH26 had high primary cytotoxicity which decreased after a week; AH Plus on the other hand had considerable cytotoxicity during the first 4 hours only (4). Cytokines are glycoproteins which are produced during inflammatory processes (1). Dysregulated cytokine productions at local sites have been considered to be major contributors to the development of inflammatory diseases (5). Interleukin-6 (IL-6) and IL-8 released have been reported to play an important role in the pathogenesis of inflammation (5). However, there is little information about the precise mechanism of root canal sealer-induced inflammatory reaction. IL-6 has a dual effect; in acute inflammation it can have a protective effect but in chronic inflammation it is somewhat pro-inflammatory (6). Since in apical periodontitis we encounter a chronic situation, the release of IL-6 by mononuclear cells or fibroblast could be destructive.

The aim of the present *in vitro* study was to compare the cytotoxic effects of 4 sealers with different bases (Tubliseal EWT, Sankin, Apexit and AH Plus) on rat L929 fibroblasts. Also, the release of IL-6 cytokine from fibroblasts was evaluated to assess the inflammatory effects of the sealers.

## MATERIALS AND METHODS

L929 rat fibroblasts were obtained from Pasteur Institute, Tehran, Iran. Cells were grown in complete medium culture (CMC) supplemented with 10% fetal bovine serum (FBS) (Gibco, US) and 100 µg/mL penicillin and 100 µg/mL Streptomycin (Sigma Co., US) under standard cell culture condition (37˚C, 100% humidity, 95% air, 5% CO_2_). Cells were used in this study after the fourth passage. Experimental groups were as follow: group 1, cells were treated with sealers immediately after the mixing; group 2, cells were treated with sealers 3 hours after the mixing. Each group had 5 subgroups including subgroup1: AH plus (Dentsply, DeTrey, Germany), subgroup 2: Sankin (Sankin, Kogyo, K.K., Japan), subgroup 3: Apexit (Vivadent Schann Vaduz, Liechtenstein), subgroup 4: Tubliseal EWT (Kerr Co., Romulus, MI, USA), subgroup 5: no treatment (control).

For material preparation, tubes with 0.7 internal diameters were cut into lengths of 5 mm each, submerged in distilled water for 24 hours and sterilized at 121˚C for 20 minutes. Materials were mixed according to manufacturer's instruction in sterilized conditions under a laminar air flow hood. The tubes were filled with sealers and all procedures were done on vibrator (Delta, Germany). In the first group, the samples were added to cell wells immediately after mixing and in the second group, the samples were added 3 hours after mixing. During these three hours, they were kept under UV-light hood.

After 1 h, 24 h, and 7 days of incubation, cell viability was evaluated by 3-(4,5 dimethyl-thiazol-2-yl)-2,5-diphenyltetrazolium bromide (MTT) assay according to the manufacturer's instruction (Merck Co, Darmstadt, Germany).

For inflammatory evaluation of sealers IL-6 level was measured in group two. The cultured suspension was collected and preserved in micro tubes. Micro tubes were cryopreserved at -20˚C during the experiment. For determination of fibroblast cytokine level, IL-6 kit (Bender Med System, Vienna, Austria) was used. Each well of ELISA plate was filled with anti IL-6-monoclonal antibody, and then samples of IL-6 were conjugated with biotin and were added to wells and kept for 2 hours at room temperature. The samples were rinsed with distilled water in order to eliminate unbounded compounds. Streptavidin HRP was added in order to be bonded with conjugated biotin-interleukin. Rinsing was carried out again after 1 hour at room temperature and samples were assessed at 450 nm. The quantity of staining was directly related to the concentration of IL-6.

Data were analyzed using Kruskal-Wallis and Mann-Whitney U tests.

## RESULTS

**Figure 1:**
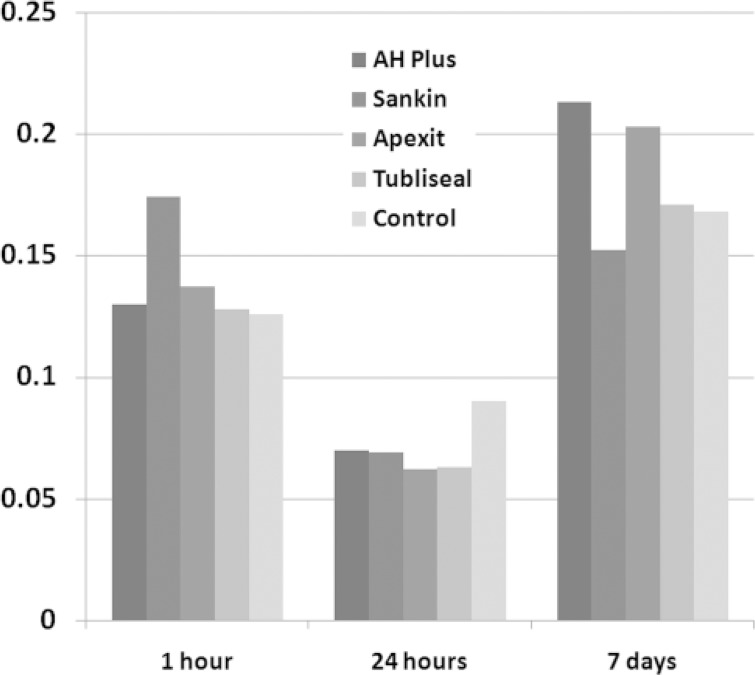
Comparison of optical density (OD) of viable cells after exposure to 4 different sealers in first group (fresh specimen)

**Figure 2:**
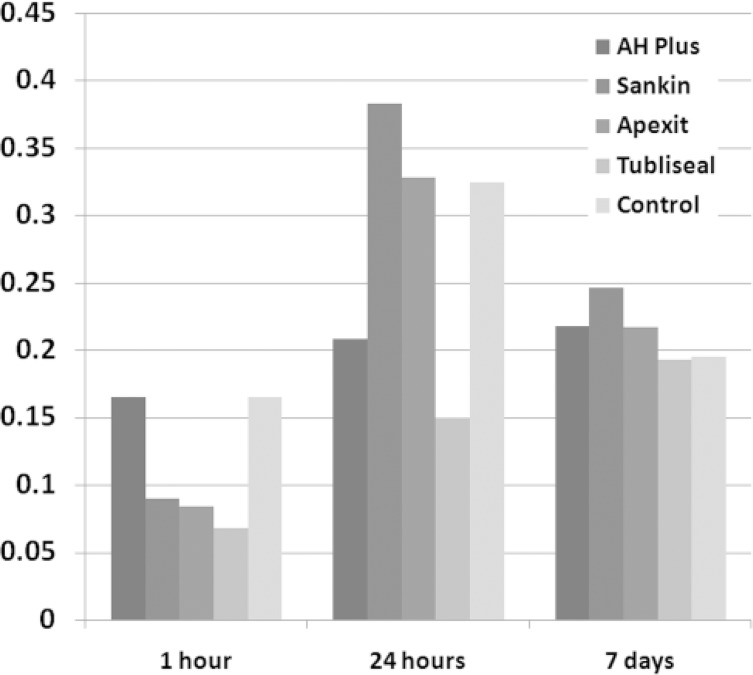
Comparison of optical density of viable cells after exposure to 4 different sealers in second group (set specimen)

Significant differences were observed in cytotoxicity of different sealers in both groups (P<0.001). In group 1 (fresh specimens), the lowest cytotoxicity belonged to Sankin with a minor and insignificant increase. In group 2 (set specimens) the lowest cytotoxicity belonged to AH Plus which increased after 24 h, decreased after 7 days and finally reached the control group level. Figure 1 and Figure 2 have summarized the findings. They show viability of fibroblast after exposure to various sealers in both groups. Regarding the production of IL-6, significant differences were seen among the sealers (P<0.001). The highest level was observed in Sankin followed by Tubliseal EWT, and the lowest level was achieved in AH Plus group (Table 1).

## DISCUSSION

Fibroblasts are the major cells of connective tissue and have the ability to produce and protect connective matrix. During the inflammatory phase, the fibroblasts stimulated by inflammatory cytotoxin and bacterial products take part in connective tissue lysis (7). Although different cells are used for cytotoxic evaluation; one of the most common is rat L929 fibroblasts (2,4,8). This cell line is easy to prepare and culture without the individual difference of primary cells. In this study, fibroblasts were used in monolayer form. However other studies did not demonstrate significant cytotoxic differences between mono and multilayer cells (9).

The MTT test was used for cytotoxic evaluation. This *in vitro* study is a relatively simple, reproducible and accurate; also a radioisotope is not required.

Ersev and colleagues evaluated the cytotoxic effects of set sealers (Traitment Ketac Endo, CRCS, Tubliseal, Endomethasone and AH26) and claimed that the cytotoxicity of Tubliseal remained high after 1 week and increased with time, a finding that concurs with our observation. It would be wise to mention that the main ingredients of Tubliseal EWT and Tubliseal are the same (10). Leonardo *et al.* evaluated the cytotoxic effects of Apexit, CRCS, and Sealapex on rat's peritoneum macrophage morphology and concluded that Apexit has the highest cytotoxicity; this may be the result of inadequate adaptation and dissolving. Interestingly our analysis showed the same results. In all studies of cytotoxicity it is necessary to change cultures in order to eliminate cellular metabolites and provide new media for cell nutrition; hence materials concentrations decreased during these procedures (11). This may be have resulted in the decrease of cytotoxicity after 7 days.

**Table 1:** Comparison of fibroblast IL-6 production after exposure to 4 different sealers in group2 (set specimen)

**Sealers**	**Mean ± SD (ng/ml)**
**AH Plus**	0.060 ± 0.0035
**Sankin**	0.070 ± 0.0012
**Apexit**	0.060 ± 0.0036
**Tubliseal**	0.065 ± 0.0050
**Control**	0.089 ± 0.0440

Camps *et al.* in an investigation of cytotoxicity of AH Plus, Cortisonol and Sealapex on L929 rat's fibroblasts concluded that AH plus had the lowest cytotoxicity from the first day up to the third day; our study on the other hand demonstrated the lowest cytotoxicity on the 7^th^ day. They utilized set specimens only for evaluation, on the other hand, we did not evaluate the cytotoxicity in 48 and 72 hours intervals (12).

Haung *et al.* assessed the cytotoxicity of set sealers (AH26, AH plus, N2 canals, Endomethasone, Sealapex) on human PDL and V19-Hamster cells and concluded that AH plus causes the inhibition of PDL cells’ growth from the first day up to the third day. The cytotoxicity chiefly decreased from the second day (13). Their results are in agreement with our observations.

To thoroughly investigate the inflammatory effects of sealers various methods can be employed, for example assessing different immunologic cells as well as the numerous cytokines that are released from inflammatory cells. Since measurement of IL-6 by ELISA technique is a simple and feasible method and IL-6 is a proinflammatory marker, we decided to measure its release from fibroblast for its inflammatory effect. In this study, we measured IL-6 in group 2 only and after 24 hours. The release of sealer contents into culture medium could increase the turbidity of the samples and interfere in ELISA reading. Thus only set specimens were utilized for IL measurement. Also the release of cytokines is induced after several hours and decrease after 24 hours consequently we did not consider measurements after 1 hour and 7 days periods. It is worthy to mention Menden *et al’s *study which evaluated inflammatory and cytotoxic effects of freshly mixed and set sealers (Endofill, Sealer EWT, Kerr pulp canal) on macrophage cells. They demonstrated that both groups had the same inhibiting activity on cells growth and that the inflammatory effects (release of IL-2) were not affected by sealers. However we cannot compare their results as different cytokines, samples and cells were employed (14).

## CONCLUSION

The results of the present study indicate that AH plus and Sankin have low cytotoxicity. Interestingly, the highest amount of IL-6 production belongs to Tubliseal EWT and Sankin. Based on the results of the present study, we can conclude that Tubliseal EWT has higher inflammatory and cytotoxic effects compared to other groups.
